# Antimicrobial Effect of Calcium Hydroxide Combined with Electrolyzed Superoxidized Solution at Neutral pH on *Enterococcus faecalis* Growth

**DOI:** 10.1155/2021/6960143

**Published:** 2021-11-09

**Authors:** Héctor Armando Jimenez-Gonzalez, María Argelia Akemi Nakagoshi-Cepeda, Sergio Eduardo Nakagoshi-Cepeda, Víctor Hugo Urrutia-Baca, Myriam Angélica De La Garza-Ramos, Juan Manuel Solis-Soto, Ricardo Gomez-Flores, Patricia Tamez-Guerra

**Affiliations:** ^1^Universidad Autónoma de Nuevo León, Facultad de Odontología, Av. Dr. Eduardo Aguirre Pequeño, Mitras Centro, Monterrey, Nuevo León, C.P. 64460, Mexico; ^2^Universidad Autónoma de Nuevo León, Facultad de Ciencias Biológicas, Departamento de Microbiología e Inmunología, Laboratorio de Inmunología y Virología, Ciudad Universitaria, San Nicolás de los Garza, Nuevo León, CP 66451, Mexico; ^3^Centro de Investigación y Desarrollo en Ciencias de la Salud, Unidad de Odontología Integral y Especialidades, Av. Dr. Aguirre Pequeño y Silao S/N, Mitras Centro, Monterrey, Nuevo León, CP 64460, Mexico

## Abstract

**Objective:**

To evaluate the effect of the combination of calcium hydroxide (Ca(OH)_2_) and a novel electrolyzed superoxidized solution at neutral pH, known as OxOral® on *Enterococcus faecalis* growth in root canals.

**Methods:**

Sixty human teeth were used, from which root canals were infected and randomly divided into the following treatment groups: saline solution, saline solution plus Ca(OH)_2_, OxOral®, and OxOral® plus Ca(OH)_2_.

**Results:**

A permanent reduction in bacterial growth was observed at days 1, 6, 12, and 18 after OxOral® plus Ca(OH)_2_ treatment from 4.4 ± 0.074 log_10_ CFU/mL to 0.0 ± 0.001 log_10_ CFU/mL. In addition, alkaline conditions maintenance was observed from application time (pH = 12.2 ± 0.033) to 18 d posttreatment (pH = 12.6 ± 0.083).

**Conclusion:**

The combination of OxOral® and Ca(OH)_2_ provides an alkaline pH and inhibits *E. faecalis* growth into the root canals. Our study opens the possibility for further research on the use of OxOral® in endodontic therapy.

## 1. Introduction

Untreated dental caries, crown, or root fractures of the tooth, conservative dental treatments that have not been properly applied, and exposure of pulp tissue to chemical agents or dental materials may lead to inflammation of the dental pulp tissue, known as pulpitis. Its progression leads to an irreversible condition, where the inflammation spreads to the tooth apices, periodontium, dentinal tubules, and blood vessels [1].

The endodontic treatment is commonly recommended, when there is irreversible pulpitis or pulp necrosis. Therapy consists of the total removal of the dental pulp and the three-dimensional sealing of the root canal to restore the function of the teeth within the masticatory apparatus [2, 3]. During this treatment, the root canal is provisionally sealed with calcium hydroxide (Ca(OH)_2_), which may be dissolved in saline solution or anesthesia, to keep clean and aseptic [3–5]. Ca(OH)_2_ is an odorless white powder obtained by calcination of calcium carbonate and its transformation into calcium oxide, which possesses a molecular weight of 74.08 g/mol, low solubility in water with a pH = 12.5 − 12.8, and is insoluble in alcohol. Many studies have demonstrated the properties of Ca(OH)_2_-based sealants, such as physical properties, biocompatibility, leaks, adhesion, antibacterial, and periapical healing effects [6, 7]. The two most important reasons for using Ca(OH)_2_ in a dressing or intracanal medication are stimulation of the periapical tissues to maintain health and promotion of healing and its antimicrobial effects. Although the exact mechanisms are unknown, the following ones have been proposed: (a) Ca(OH)_2_ is antibacterial depending on the availability of free hydroxyl ions that encourages repair and active calcification [8–10]. Furthermore, it has been suggested that it acts indirectly by obliterating the root canal space, thus limiting the use of nutrients by the microorganisms lodged in the dentin [11], (b) ?added value="t"?>he alkaline pH neutralizes lactic acid from osteoclasts and prevents dissolution of mineralized components of teeth and also activates alkaline phosphatase and calcium-dependent adenosine triphosphatase reaction leading to hard tissue formation [12, 13], (c) it denatures proteins found in the root canal and makes them less toxic,and (d) it diffuses through dentinal tubules and may communicate with the periodontal ligament space to arrest external root resorption and accelerate healing [12, 13].

Despite its advantages, Ca(OH)_2_ endodontic treatments failure in teeth has been reported. *Enterococcus faecalis* has been reported as the bacterial microorganism responsible for the failure of endodontic therapies [14]*. Enterococcus faecalis*colonizes inside dentinal tubules up to 300 microns, a site that is difficult to access for the action of Ca(OH)_2_, affecting its effectiveness in endodontic therapy [15, 16]. Combining Ca(OH)_2_ with an appropriate solvent may increase its solubility, antimicrobial activity, and access to complex root canal sites. The solvent used for Ca(OH)_2_ influences both the physical and chemical properties of the material, including its viscosity and ion release pattern. A range of water-based materials has traditionally been used but there are alternative vehicles [17]. This study proposes using electrolyzed superoxidation solution (ESS) with neutral pH as the Ca(OH)_2_ solvent for the endodontic treatment. ESS has a neutral pH (6.4–7.5) and is produced from the electrolysis of sodium chloride (NaCl) solution in an electrolytic cell where a diaphragm (partition or membrane) separates the anode and the cathode. Cations are drawn towards the electrode negative, where they receive electrons, forming a negatively charged antioxidant solution (alkaline solution). At the positive electrode, anions are attracted, which give up their additional electrons to create a positively charged oxidant solution (acid solution) [18]. For ESS formation, part of an antioxidant solution formed is channeled back into the anode chamber, thus increasing the content of hypochlorite ions (OCl^−^).The reintroduction of the alkaline solution back into the acidic solution allows adjusting the pH up to neutral. Therefore, ESS is mainly composed of hypochlorous acid (HOCl), OCl^−^, chlorine (Cl^−^), and sodium hypochlorite (NaOCl). Electrolysis may produce H^+^ and OH ions, H and OH radicals, H_2_, O_2_, HO_2_, and O_3_ due to redox reactions. As a result, hydrogen and ozone gas are released, and a percentage of hydroxides remains in the solution in various forms, including but not limited to hydrogen peroxide (H_2_O_2_) [19, 20].

ESS has become a new alternative in tissue asepsis in different areas of medicine and the food industry for its high antimicrobial activity, stability, and safety [21, 22]. Some reports suggest that the presence of Cl^−^ and a high concentration of oxidation-reduction potential (ORP) are responsible for the antimicrobial activity of ESS. However, other studies suggest that HOCl is the most active of compounds [23, 24].

A significant antimicrobial activity of ESS in removing biofilms from *E. faecalis* in root canals has been previously reported [25–29]. The aim of the present study was to evaluate the antimicrobial effect of combining Ca(OH)_2_ with an ESS with neutral pH called OxOral®, in *E. faecalis*-infected root canals.

## 2. Materials and Methods

### 2.1. *E. faecalis culture conditions*

One cryovial containing *E. faecalis* (ATCC® 29212™) cells was rapidly thawed in a prewarmed 37 °C water, and cells were placed into 100 mL brain heart infusion broth (BHI; Oxoid Limited, Hampshire, UK) supplemented with 5 *μ*g/mL hemin and 0.5 *μ*g/mL menadione at 0.5 McFarland. The culture was incubated at 37 °C for 24 h under anaerobic conditions. Afterwards, 1 mL of the preculture of *E. faecalis* was collected, placed in 100 mL of BHI-supplemented broth, and incubated until reaching the exponential, logarithmic phase.

### 2.2. Instrumentation and Preparation of Teeth

Sixty human maxillary 2^nd^ premolar teeth were obtained from human volunteers of the Endodontic Postgraduate Program of the School of Dentistry at the Autonomous University of Nuevo Leon. The study was conducted following the Declaration of Helsinki, the Institutional Ethics Committee approved the protocol (SPSI-01613/00249), and informed consent was obtained from each volunteer. The dental crowns were sectioned with a diamond disc and press. The working length was determined with a #15 dental file (K-Flex) of 25 mm; 1 mm was subtracted from its exit from the apical foramen. Instrumentation began with Triple-Flex Files Stainless Steel files (SybronEndo, Ormex, Yucatan, Mexico) up to file #40 using 5.2% NaOCl as an irrigating agent between each file change to maintain conduit permeability.

The apical region of each tooth was sealed with composite resin and externally with a layer of nail varnish, excluding the cervical opening, which was enlarged with Gates GLIDDEN files to be later autoclaved at 121°C for 15 min in 1.5 mL tubes. All root portions and materials used (cotton pellets, Eppendorf tubes, instruments, files, syringes, rotary drills, diamond disc, and serrated dissection forceps) were autoclaved.

### 2.3. Contamination and Treatment of Root Canals

Each root canal was inoculated with 15 *μ*L of *E. faecalis* with a bacterial concentration of 5 log_10_ CFU/mL and incubated in an anaerobic atmosphere at 37 °C for 21 d to achieve the formation of *E. faecalis* biofilms within the root canal, after which 10 *μ*l of supplemented BHI broth were added every two days to avoid teeth dehydration and promote bacterial growth.

After incubation, 12 samples were randomly selected and plated on BHI agar to verify contamination of the root canals by CFU counting.

Sixty root canals were randomly divided into four groups, and 10 *μ*L of the treatment was applied within the root canal based on the following treatment groups: group 1, 0.9% NaCl; group 2, 0.9% NaCl plus Ca(OH)_2_ paste; group 3, OxOral®; and group 4, OxOral® plus Ca(OH)_2_ paste. Ca(OH)_2_ medication pastes were prepared by mixing 0.5 g of Ca(OH)_2_ powder plus three drops of saline solution (0.9% NaCl) or OxOral®. All treated teeth were incubated for up to 18 d.

### 2.4. Evaluation of Bacterial Growth

For *E. faecalis* growth evaluation, aliquots of 100 *μ*L were taken at days 1, 6, 12, and 18 post-treatment, serially diluted, and plated onto BHI-supplemented agar using a glass spreader. Next, plates were incubated at 37 °C for 24 h, and CFU/mL were calculated; all measurements were performed in triplicate.

### 2.5. Measurement of pH Variation

For pH measurements, 60 tubes (15 mL) containing 2 mL of BHI-supplemented broth were inoculated with 10 *μ*L of *E. faecalis* cells at ~5 log_10_ CFU/mL at 37 °C for 24 h. Tubes were then randomly divided into four groups and 9 mL of the different treatments were added. pH values were measured at days 1, 6, 12, and 18 post-treatment, by a digital pH meter (Corning®, Model 313; NY, USA) calibrated with buffer solutions (J.T.Baker-Avantor Performance Materials, S.A. de C.V, Mexico City, Mexico; pH = 4.00; pH = 7.00; and pH = 10.00) before each experiment. After removing the specimens, the container was placed in an orbital shaker (Prendo, INO-650 M, Mexico City, Mexico) for 5 sec before measuring. The room temperature during the test was 25 °C.

### 2.6. Statistical Analysis

Results were expressed as means ± standard deviation (SD) of the response of three replicate determinations per treatment from three independent experiments. The significance level was assessed by the ANOVA and Tukey's tests (*p* < 0.05), using the SPSS statistics software version 22.

## 3. Results

### 3.1. Evaluation of Antimicrobial Properties of OxOral® in the Root Canals

*E. faecalis* growth was determined at days 6, 12, and 18. One-way ANOVA test was performed, taking the bacterial growth (log_10_ CFU/mL) as the dependent variable and the treatment groups and the time as independent variables. The results show significant differences in the treatment groups and the time, with a *p* = 0.001 and *p* = 0.012, respectively.

Our results showed significant (p < 0.05) antimicrobial activity in the root canals treated with saline solution plus Ca(OH)_2_, OxOral®, and OxOral® plus Ca(OH)_2_ at 24 h with 0.051 ± 0.072, 0.044 ± 0.07, and 0.000032 ± 0.072 log_10_ CFU/mL, respectively. In addition, a sustained inhibitory effect was observed in saline solution plus Ca(OH)_2_ and OxOral® until day 6 posttreatment. However, only the combination of OxOral® plus Ca(OH)_2_ maintained its inhibitory potential against *E. faecalis* after 18 d post-treatment, as compared with the other treatments (*p* < 0.05), whereas saline solution showed a progressive increase in bacterial growth from 4.55 ± 0.071 log10 CFU/mL (day 0 post-treatment) to 10.05 ± 0.075 (day 18 post-treatment), as expected(Table 1).

### 3.2. pH Measurements in the Treated Root Canals

The pH variation of the different treatments at days 0, 1, 6, 12, and 18 was also evaluated. One-way ANOVA test was performed, where the test variable was pH in function of the treatment groups and time. We observed a statistically significant (p < 0.05) difference only in the treatment group but no difference was observed with regard to time (*p* > 0.05).

pH measurements in the root canals treated with saline solution and OxOral® showed neutral pH maintenance from day 0 (baseline) with 6.25 ± 0.075 and 7.003 ± 0.321 until day 18 post-treatment with 7.41 ± 0.291 and 7.456 ± 0.196, respectively, whereas root canals treated with saline solution plus Ca(OH)_2_ and OxOral® plus Ca(OH)_2_ showed an alkaline pH from day 0 (baseline) with 12.149 ± 0.021 and 12.22 ± 0.033 until day 18 with 12.522 ± 0.02 and 12.586 ± 0.083, respectively. We did not observe statistical differences in pH values between these treatment groups (Table 2).

### 3.3. Consistency of OxOral® plus Ca(OH)_2_ Combination

**Figure 1:**
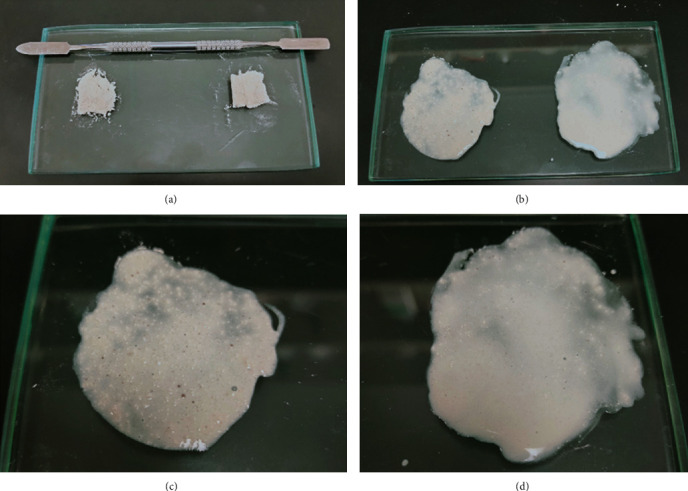
Comparison of the Ca(OH)_2_ solvents. (a) Comparison of the amount of Ca(OH)_2_ powder used; (b) comparison of the macroscopic characteristics of the combination of saline solution plus Ca(OH)_2_ (left) and OxOral plus Ca(OH)_2_ (right); (c) combination of saline solution plus Ca(OH)_2_; (d) combination of OxOral plus Ca(OH)_2_.

Figure 1 shows a comparison of the macroscopic characteristics of the combination of saline solution plus Ca(OH)_2_ and OxOral® plus Ca(OH)_2_, where we observe a higher solubility and pasty consistencies compared with the traditional combination.

## 4. Discussion

One of the requirements of an endodontic root canal sealer is that it should not be cytotoxic and immunologically compatible with peripheral tissue. Therefore, a specific root canal sealer biocompatibility and antimicrobial activity remain one of the major considerations for selecting an appropriate sealer for a dental restoration. Ca(OH)_2_ has been widely used since the 1920s for its biocompatibility, antimicrobial potential, and ftissue restoration support in endodontic therapies [30].

Hydroxyl ions are responsible for Ca(OH)_2_ antimicrobial activity.Therefore, the efficacy of any product varies according to the availability of these ions in solution, which in turn reflects the nature of the solvent used. Most existing commercial Ca(OH)_2_ use vehicles of water. However, other agents have recently been mixed with water, such as glycerin or polyethylene glycol (PEG) [17].

The pH values from using OxOral are similar to some commercial Ca(OH)_2_ products that use vehicles of water such as Calasept Plus™, Calcipulpe™, DT Temp™, Pulpdent™, and Ultracal XS™ with pH values between 11.8 and 12.7 [17, 31]. However, there are reports where the pH reaches up to 15.0 in Ca(OH)_2_ products, where a mixture of water with PEG is used, such as Calmix™ [32].

There are previous studies, where the use of neutral pH solutions is reported as a vehicle for the root canal Ca(OH)_2_ pastes. For example, Tronstad et al. used a Ringer's solution (0.125 M NaCl, 1.5 mM CaCl_2_ dihydrate, and 5 mM KCl; pH = 7.3 − 7.4) combined with Ca(OH)_2_ and filled the root canal of green monkey teeth (*Cercopithecus aethiops sabaeus*), and they observed maintenance of alkalinity conditions with a pH = 12.2 after four weeks [33]. However, there are no reports on using ESS at neutral pH as a vehicle to prepare Ca(OH)_2_.

Some studies suggest that Ca(OH)_2_ is a slow-acting antimicrobial, which requires at least 24 hours to produce a bactericidal effect against *Enterococci* spp. Furthermore, Ca(OH)_2_ hydroxide hydrolyzes the lipid moiety of bacterial LPS, causing its biological inactivation, the desired effect to prevent an inflammatory reaction in the periapical tissue [34, 35].

For many years, different compounds have been evaluated for root canal disinfection, Ca(OH)_2_-based pastes being the most widely used.However, the permanence in time of these pastes is one of the main discrepancies between the authors, mainly in the cases of the teeth with pulp necrosis and periapical lesion [36, 37]. Our study observed the intracanal permanence of the Ca(OH)_2_ plus saline solution or OxOral® pastes for up to 18 d.

Root treatment consists of debriding and disinfecting the entire root canal system, which requires eliminating the pulp tissue and the microorganisms causing the infection, adopting instrumentation and chemical irrigation mechanisms, and subsequently a medication in the root canal between treatment sessions. The success of endodontic treatment depends on the removal of microbes from the root canals and avoidance of reinfection.

Paudel et al. found that 0.9% NaCl was as effective as sequential use of 3% H_2_O_2_, 5.2% NaOCl, and 0.9% NaCl, although saline has no antibacterial activity [38]. In addition, it has been reported that the exposure and contact time during the irrigation of human root canals are crucial, since treatment with ozonized water, 2.5% NaOCl, 2% CHX, and gaseous ozone for 20 min was not sufficient to inactivate *E. faecalis* [39]. Therefore, in our study we evaluated the antimicrobial activity of treatments from one to 18 d post-treatment.

With a significant amount of calcium carbonate, the Ca(OH)_2_ paste has a granular consistency due to its low solubility (1.73 g/L at 20 °C). One of the most interesting observations in the present study was the ease of removing the paste composed of Ca(OH)_2_ plus OxOral®, unlike that composed of Ca(OH)_2_ plus saline solution. However, other studies are necessary to evaluate any physical-chemical changes.

On the other hand, reactive oxygen and chlorine species are derived during the electrolysis process to produce electrolyzed water and have been previously evaluated in endodontic treatment. In this regard, Zhou et al. reported that a plasma jet with or without helium flowing through 3% hydrogen peroxide effectively sterilized root canals infected with *Enterococcus faecalis* [40]. Another study conducted by Mihadi et al. assessed the cytocompatibility and antibacterial activity of different concentrations of CHX combined with H_2_O_2_ compared with the action of 5.25% and 2.5% NaOCl. All combinations of CHX and H_2_O_2_ except 0.1% CHX plus 3% H_2_O_2_ were efficient irrigants against planktonic *E. faecalis* and had a better cytocompatibility with PDL cells than 5.25% and 2.5% NaOCl [41].

Some studies have reported a significant antimicrobial activity of electrolyzed superoxidized water [42, 43]. Different antimicrobial broad-spectrum disinfectants are manufactured by Esteripharma®, Mexico, SA of CV, each with different compositions and proposes. Velázquez-Meza et al. evaluated the antimicrobial activity based on superoxidized water called Estericide Qx® against 524 clinical bacterial isolates causing nosocomial infections, including Gram-negative (*Escherichia coli* and *Pseudomonas aeruginosa* beta-lactam resistant) and Gram-positive (*Staphylococcus aureus* and *Streptococcus epidermidis* methicillin-resistant, and *Enterococcus faecium*). The results showed that Estericide Qx® provides a broad-spectrum antibacterial activity mainly in Gram-negative [44]. Other studies, Lucio-Sauceda et al., reported a significant anti-*H. pylori* activity of OxOral® by microdilution assays, and Rivera-Garcia et al. observed a significant antimicrobial activity against *Listeria monocytogenes* on eggshells [18, 22].

Landa-Solis et al. treated pure cultures of *S. aureus*, *E. coli*, *P. aeruginosa*, *Salmonella typhi*, and *Candida albicans* with Microcyn® and found it was active on all microorganisms tested [45]. In addition, Vorobjeva et al. reported that superoxidized solution was effective on spores, Gram-positive, and Gram-negative bacteria related to nosocomial infections [46].

According to many studies, chlorine and a high concentration of ORP in ESS seem to be responsible for its antimicrobial activity. Active chlorine compounds destroys the membranes of microorganisms. Other modes of chlorine action (e.g., decarboxylation of amino acids, reactions with nucleic acids, and unbalanced metabolism after the destruction of crucial enzymes) also have been proposed [20, 47]. Studies suggest that HOCl is the most active of the chlorine compounds because HOCl penetrates cell membranes and produces hydroxyl radicals, which exert their antimicrobial activity through the oxidation of crucial metabolic systems. In addition, OH radicals, which are the most potent oxidizing agents, also have shown antimicrobial activity [24, 29, 48].

The main characteristics of Ca(OH)_2_ are its limited solubility, high pH, and use as a broad-spectrum antimicrobial agent. Despite the benefits and advantages of Ca(OH)_2_, its use is cumbersome. Proper handling and placement are challenging for the dentist. In addition, its removal from the canal is usually incomplete, with 20-45% residue on the canal wall, even after copious irrigation using saline, sodium hypochlorite, or EDTA. Residual Ca(OH)_2_may shorten the setting time of zinc oxide-eugenol endodontic sealants. In particular, it may interfere with the sealing of a root filling and affect the quality of the treatment.

On the other hand, the potential of Ca(OH)_2_ to eliminate bacteria from the root canal has recently been challenged. Some *in vitro* studies have reported that dentin inactivates the antimicrobial activity of Ca(OH)_2_, and a clinical study reported an increase in bacterial growth after its application. Our findings agree with these studies since the combination of Ca(OH)_2_ plus saline solution showed an increase in bacterial growth of *E. faecalis* after day six of treatment. However, in the root canals treated with the combination of Ca(OH)_2_ plus OxOral®, we did not observe *E. faecalis* growth. Therefore, it is possible that OxOral® may have a synergistic effect with Ca(OH)_2_.

## 5. Conclusions

Taken together, the results of the present study demonstrated that the combination of OxOral® plus Ca(OH)_2_ showed a significant antimicrobial activity and solubility of Ca(OH)_2_, prolonging the alkaline pH conditions. Our results open the possibility for further research on the use of OxOral® for sealing root canals, during endodontic treatment.

## Figures and Tables

**Table 1:** *Enterococcus faecalis* growth in the presence of 0.9% sodium chloride, 0.9% sodium chloride plus calcium hydroxide, OxOral, and OxOral plus calcium hydroxide.

Posttreatment time (days)	Treatment groups (log_10_ CFU/mL ± SD^∗^)			
	0.9% NaCl	0.9% NaCl + Ca(OH)_2_	OxOral	OxOral + Ca(OH)_2_
0	4.55 ± 0.071	4.47 ± 0.07	4.6 ± 0.074	4.443 ± 0.068
1	8.02 ± 0.069	0.051 ± 0.072	0.044 ± 0.07	0.000032 ± 0.072
6	10.01 ± 0.07	0.1 ± 0.071	0.402 ± 0.071	0.0000031 ± 0.074
12	10.02 ± 0.073	1.201 ± 0.074	0.993 ± 0.07	0.00005 ± 0.074
18	10.05 ± 0.075	2.021 ± 0.071	1.904 ± 0.075	0.00003 ± 0.07

^∗^The data represent mean ± standard deviation of log_10_ CFU/mL.

**Table 2:** pH values in the presence of 0.9% sodium chloride, 0.9% sodium chloride plus calcium hydroxide, OxOral, and OxOral plus calcium hydroxide.

Posttreatment time (days)	Treatment groups (mean pH ± SD^∗^)			
	0.9% NaCl	0.9% NaCl + Ca(OH)_2_	OxOral	OxOral + Ca(OH)_2_
0	6.25 ± 0.075	12.149 ± 0.021	7.003 ± 0.321	12.22 ± 0.033
1	6.606 ± 0.092	12.268 ± 0.024	7.048 ± 0.4	12.448 ± 0.05
6	6.606 ± 0.092	12.268 ± 0.02	7.048 ± 0.429	12.448 ± 0.05
12	6.606 ± 0.09	11.794 ± 0.656	6.506 ± 0.495	12.272 ± 0.130
18	7.41 ± 0.291	12.522 ± 0.02	7.456 ± 0.196	12.586 ± 0.083

^∗^The data represent mean ± standard deviation of pH measurements.

## Data Availability

The data used to support the findings of this study are available from the corresponding author upon request.

## References

[1] Cushley S., Duncan H. F., Lappin M. J. (2019). Pulpotomy for mature carious teeth with symptoms of irreversible pulpitis: a systematic review. *Journal of Dentistry*.

[2] Morotomi T., Washio A., Kitamura C. (2019). Current and future options for dental pulp therapy. *Japanese Dental Science Review*.

[3] Lee B.-N., Moon J.-W., Chang H.-S., Hwang I.-N., Oh W.-M., Hwang Y.-C. (2015). A review of the regenerative endodontic treatment procedure. *Restorative Dentistry & Endodontics*.

[4] Yazdanpanahi N., Behzadi A., Zare Jahromi M. (2021). Long-term pH alterations in the periradicular area following the application of calcium hydroxide and MTA. *Journal of Dentistry*.

[5] Estrela C., Holland R., Estrela C. R. . A., Alencar A. H. G., Sousa-Neto M. D., Pécora J. D. (2014). Characterization of successful root canal treatment. *Brazilian Dental Journal*.

[6] Xie Z., Shen Z., Zhan P. (2021). Functional dental pulp regeneration: basic research and clinical translation. *International Journal of Molecular Sciences*.

[7] Desai S., Chandler N. (2009). Calcium hydroxide-based root canal sealers: a review. *Journal of Endodontics*.

[8] Singh S., Srivastava B., Gupta K., Gupta N., Singh S., Singh S. (2020). Comparative evaluation of antifungal efficacy of five root canal sealers against clinical isolates of candida albicans: a microbiological study. *International Journal of Clinical Pediatric Dentistry*.

[9] Arandi N. (2017). Calcium hydroxide liners: a literature review. *Clinical, Cosmetic and Investigational Dentistry*.

[10] Cvek M., Mejare I., Andreasen J. O. (2004). Conservative endodontic treatment of teeth fractured in the middle or apical part of the root. *Dental Traumatology*.

[11] Ørstavik D. (2003). Root canal disinfection: a review of concepts and recent developments. *Australian Endodontic Journal*.

[12] Athanassiadis B., Abbott P. V., Walsh L. J. (2007). The use of calcium hydroxide, antibiotics and biocides as antimicrobial medicaments in endodontics. *Australian Dental Journal*.

[13] Estrela C., Decurcio D. . A., Rossi-Fedele G., Silva J. A., Guedes O. A., Borges Á. H. (2018). Root perforations: a review of diagnosis, prognosis and materials. *Brazilian Oral Research*.

[14] Alghamdi F., Shakir M. (2020). The influence of Enterococcus faecalis as a dental root canal pathogen on endodontic treatment: a systematic review. *Cureus*.

[15] Vatkar N. A., Hegde V., Sathe S. (2016). Vitality of Enterococcus faecalis inside dentinal tubules after five root canal disinfection methods. *Journal of Conservative Dentistry*.

[16] Kranz S., Guellmar A., Braeutigam F. (2021). Antibacterial effect of endodontic disinfections on Enterococcus faecalis in dental root canals—an in-vitro model study. *Materials*.

[17] Athanassiadis B., Walsh L. J. (2017). Aspects of solvent chemistry for calcium hydroxide medicaments. *Materials*.

[18] Lucio-Sauceda D. G., Urrutia-Baca V. H., Gomez-Flores R., De La Garza-Ramos M. A., Tamez-Guerra P., Orozco-Flores A. (2019). Antimicrobial and anti-biofilm effect of an electrolyzed superoxidized solution at neutral-pH against Helicobacter pylori. *BioMed Research International*.

[19] Ayebah B., Hung Y.-C. (2005). Electrolyzed water and its corrosiveness on various surface materials commonly found in food processing facilities. *Journal of Food Process Engineering*.

[20] Ogunniyi A. D., Dandie C. E., Ferro S. (2019). Comparative antibacterial activities of neutral electrolyzed oxidizing water and other chlorine-based sanitizers. *Scientific Reports*.

[21] You H. S., Fadriquela A., Sajo M. E. J. (2017). Wound healing effect of slightly acidic electrolyzed water on cutaneous wounds in hairless mice via immune-redox modulation. *Biological & Pharmaceutical Bulletin*.

[22] Rivera-Garcia A., Santos-Ferro L., Ramirez-Orejel J. C. (2019). The effect of neutral electrolyzed water as a disinfectant of eggshells artificially contaminated withListeria monocytogenes. *Food Science & Nutrition*.

[23] Kaczmarek M., Avery S. V., Singleton I. (2019). Microbes associated with fresh produce: Sources, types and methods to reduce spoilage and contamination. *Advances in Applied Microbiology*.

[24] Yan P., Daliri E. B., Oh D. H. (2021). New clinical applications of electrolyzed water: a review. *Microorganisms*.

[25] Zan R., Kutlu G., Hubbezoglu I., Sumer Z., Tunc T., Mutlu Z. (2015). Bactericidal effects of various irrigation solutions against Staphylococcus aureus in human root canal. *Journal of Istanbul University Faculty of Dentistry*.

[26] Herrera Saucedo A., Corona Guerra M. A., Vara Padilla F. J., Gutiérrez Valdez D. H., Alavez Rebollo S. L. (2017). Comparación de la eficacia de los irrigantes OxOral ® y NaOCl en la eliminación de Enterococcus faecalis. *Revista Odontológica Mexicana*.

[27] Briones M. E. R., Silva-Herzog Flores D., González Amaro A. M., Rodríguez R. O. (2013). Comparative assessment of the antimicrobial capacity of an electrolyzed superoxide solution of neutral pH and a hydrogen peroxide-based solution. *Revista de la Asociación Dental Mexicana*.

[28] Cheng X., Tian Y., Zhao C. (2016). Bactericidal Effect of Strong Acid Electrolyzed Water against Flow Enterococcus faecalis Biofilms. *Journal of Endodontics*.

[29] Okamura T., Tamura M., Suguro H. (2019). Bactericidal and cytotoxic effects of acid-electrolyzed functional water. *Journal of Oral Science*.

[30] Fonseca D. A., Paula A. B., Marto C. M. (2019). Biocompatibility of root canal sealers: a systematic review of in vitro and in vivo studies. *Materials*.

[31] Athanassiadis B., Abbott P. V., George N., Walsh L. J. (2010). An in vitro study of the antimicrobial activity of some endodontic medicaments against Enteroccus faecalis biofilms. *Australian Dental Journal*.

[32] Teoh Y.-Y., Athanassiadis B., Walsh L. J. (2016). The influence of aqueous and PEG 400 solvent vehicles on hydroxyl ion release from calcium hydroxide medicaments. *International Dentistry*.

[33] Tronstad L., Andreasen J. O., Hasselgren G., Kristerson L., Riis I. (1981). pH changes in dental tissues after root canal filling with calcium hydroxide. *Journal of Endodontics*.

[34] Safavi K., Nichols F. (1993). Effect of calcium hydroxide on bacterial lipopolysaccharide. *Journal of Endodontics*.

[35] Adl A., Motamedifar M., Shams M. S., Mirzaie A. (2015). Clinical investigation of the effect of calcium hydroxide intracanal dressing on bacterial lipopolysaccharide reduction from infected root canals. *Australian Endodontic Journal*.

[36] Mohammadi Z., Dummer P. M. H. (2011). Properties and applications of calcium hydroxide in endodontics and dental traumatology. *International Endodontic Journal*.

[37] Fava L. R. G., Saunders W. P. (1999). Calcium hydroxide pastes: classification and clinical indications. *International Endodontic Journal*.

[38] Paudel K. R., Jaiswal A., Parajuli U., Bajracharya M. (2011). Different pharmacological solutions in intracanal irrigation. *Nepal Medical College Journal*.

[39] Estrela C., Estrela C. R. A., Decurcio D. A., Hollanda A. C. B., Silva J. A. (2007). Antimicrobial efficacy of ozonated water, gaseous ozone, sodium hypochlorite and chlorhexidine in infected human root canals. *International Endodontic Journal*.

[40] Zhou X.-C., Li Y.-L., Liu D.-X., Cao Y.-G., Lu X.-P. (2016). Bactericidal effect of plasma jet with helium flowing through 3% hydrogen peroxide against Enterococcus faecalis. *Experimental and Therapeutic Medicine*.

[41] Mirhadi H., Abbaszadegan A., Ranjbar M. A. (2015). Antibacterial and toxic effect of hydrogen peroxide combined with different concentrations of chlorhexidine in comparison with sodium hypochlorite. *Journal of Dentistry*.

[42] Gunaydin M., Esen S., Karadag A. (2014). In vitro antimicrobial activity of Medilox® super-oxidized water. *Annals of Clinical Microbiology and Antimicrobials*.

[43] Lee S. H., Choi B. K. (2006). Antibacterial effect of electrolyzed water on oral bacteria. *Journal of Microbiology*.

[44] Velazquez-Meza M. E., Hernández-Salgado M., Sánchez-Alemán M. A. (2015). Evaluation of the antimicrobial activity of a super oxidized solution in clinical isolates. *Microbial Drug Resistance*.

[45] Landa-Solis C., González-Espinosa D., Guzmán-Soriano B. (2005). Microcyn^tm^: a novel super-oxidized water with neutral pH and disinfectant activity. *Journal of Hospital Infection*.

[46] Vorobjeva N. V., Vorobjeva L. I., Khodjaev E. Y. (2004). The bactericidal effects of electrolyzed oxidizing water on bacterial strains involved in hospital infections. *Artificial Organs*.

[47] Hricova D., Stephan R., Zweifel C. (2008). Electrolyzed water and its application in the food industry. *Journal of Food Protection*.

[48] Zeng X., Tang W., Ye G. (2010). Studies on disinfection mechanism of electrolyzed oxidizing water on E. coli and Staphylococcus aureus. *Journal of Food Science*.

